# Supplementary figures and images for: Plasma CXCL8 and MCP-1 as surrogate plasma biomarkers of latent tuberculosis infection among household contacts–A cross-sectional study
Source: PLOS Glob Public Health. 2023 Nov 22;3(11):e0002327. doi: 10.1371/journal.pgph.0002327 (PMC10664947; doi:10.1371/journal.pgph.0002327)

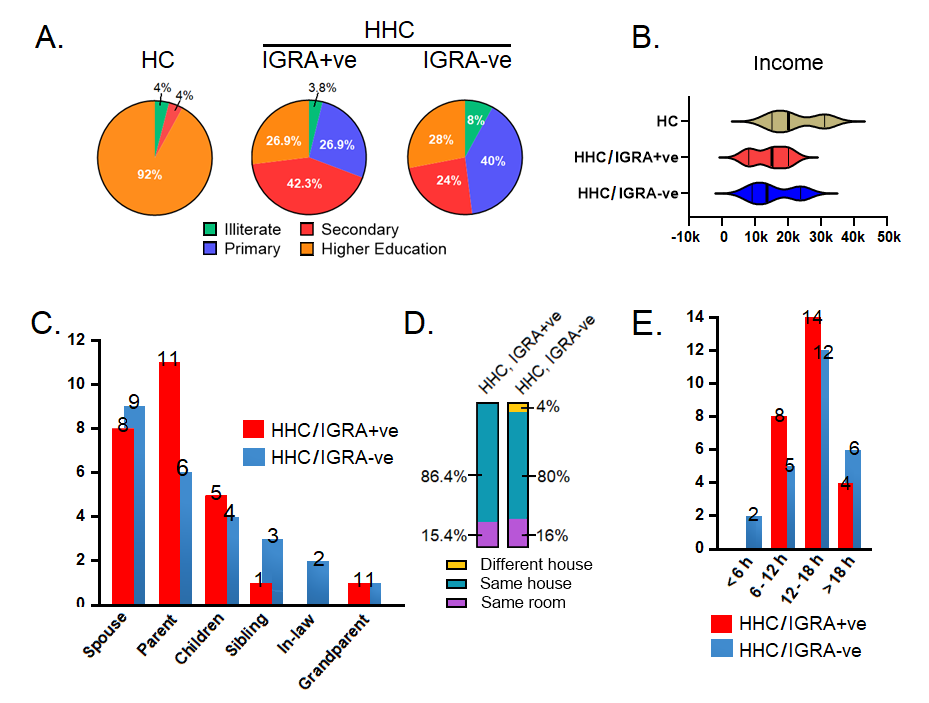

Supplement: S1 Fig — A) Education level of participants. B) Family annual income of the participants. C) Relationship of the active TB index case with the participants. D) Proximity of the participants with the active TB index case. E) Contact duration of the participants with the active TB index case. HC, healthy control; HHC, household contact; IGRA, interferon gamma releasing assay. (TIF) [file pgph.0002327.s001.tif]
