# Supplementary material for: Plasma CXCL8 and MCP-1 as surrogate plasma biomarkers of latent tuberculosis infection among household contacts–A cross-sectional study
Source: PLOS Glob Public Health. 2023 Nov 22;3(11):e0002327. doi: 10.1371/journal.pgph.0002327 (PMC10664947; doi:10.1371/journal.pgph.0002327)
Supplement: Attachment — Submitted filename: Resonse to Reviewers - PLOS Global Public Health.docx [file pgph.0002327.s002.docx]

**Response to Reviewers**

**1. Can you please clarify why you decided to perform random sampling again on the participants to select out those for Bio-Plex assay? . The information in the flow diagram in figure 1A is inconsistent with the body text i.e. you mention 26 IGRA positives yet in the flow diagram, it's all the 58 IGRA positive participants.**

**Reply:** Yes. The participants were recruited for the Bio-Plex assay by random sampling because we required a small study population from the initial group. In order to minimize the chances of biasness, random sampling was performed. The number of participants for the IGRA positive participants in the flow diagram is erroneous (Instead of 58 it should read as 26 IGRA positive). The marked error has been corrected. Thank you for pointing this out.

**2. Table 1: Participants included in all groups had underlying medical conditions. Although this was not statistically different, this could hugely confound the results. You only highlighted on page 4 in section 3.1 about the 13 IGRA +ve individuals with underlying medical conditions but in the table 1 even the healthy had underlying conditions. The participants were enrolled within a period of one week, it is not clear how and when you performed the enrolments for healthy controls – it may not matter but these individuals might not be as healthy as they indicated. They can no longer be considered healthy if they have any other underlying conditions.**

**Reply:** We agree that this would confound the results. However, we believe that random sampling would minimise any bias across all the study groups which still would have individuals with underlying conditions (that we believe would minimise the confounding effects). Thirteen individuals in LTBI, 12 individuals each in HHCs and HCs possessed underlying health conditions. The point was overlooked from mentioning under Section 3.1, which has now been incorporated in the revised version. In this study, the healthy contacts are defined as the ones who did not have TB or LTBI or any previous history of household contact with TB patients.

**3. The word 'postulate' in abstract conclusion 'We postulated that CXCL8 and MCP-1 could be the surrogate biomarkers of LTBI, especially in resource-limited settings', does not make the conclusion meaningful. Please consider rephrasing.**

**Reply:** The conclusion has now been rephrased as: “Our study suggests that CXCL-8 and MCP-1 could serve as the surrogate biomarkers of LTBI, particularly in resource-limited settings.”

-------------------------------------------------------------------------------------------------------------

**Reviewer #1:**

**1. The manuscript addresses an important topic identification of a biomarker to differentiate latent TB.**

**Reply:** Thank you for the recommendation.

**2. The manuscript requires improvement in English writing style.**

**Reply:** The language style has been improved in the revised manuscript.

**3. The title seems incomplete, consider rewriting the title**

**Reply:** The title has been modified.

**4. In the abstract the conclusion looks more of a hypothetical statement I recommend that you rewrite the conclusion.**

**Reply:** The conclusion has now been rephrased as: “Our study suggests that CXCL-8 and MCP-1 could serve as the surrogate biomarkers of LTBI, particularly in resource-limited settings.”

**5. What was the aim of the study? The aim is not clearly written**

**Reply:** The aim of the study was to identify specific biomarkers that can help diagnose LTBI and the potential risk of development of TB disease. This has now been mentioned in the revised manuscript.

**6. The introduction does show the relationship between latent TB and the CXCL8 and MCP-1 and its importance in TB immunity and hence a potential biomarker**

**Reply:** The suggestion is noted and improvements have been made in the introduction section of the revised manuscript.

**7. The results show a decrease in TNF-α, and IFN-γ. These cytokines have not been mentioned in the introduction and no relationship with the chemokines was mentioned. Furthermore the title does not reflect other cytokines**

**Reply:** Yes! The TNF-α and IFN-ɣ showed a decrease in the levels. Information about these two cytokines are added to the introduction section of the revised manuscript.

**8. The methods section does not show analysis of biochemical test however they are included in the results section.**

**Reply:** We have incorporated the biochemical parameters tested in the methods section of the revised manuscript.

**9. The ROC curve analysis is not clear figure A shows three ROC curve analyses.**

**Was the analysis between the**

**LTBI and negative house hold contacts**

**LTBI and HC**

**HHC and HC**

**Reply:** All the three ROC curves were performed between the LTBI and HCs.

**10. In your discussion you have this statement, “However, we showed that individuals with LTBI had increased plasma CXCL8 but decreased MCP-1 level” s. What is your deduction of the relationship between these two chemokines and the possible cause of the altered immunity ?**

**Is the conclusion based on the predictions mad e by the roc analysis?**

**Reply:** The increased levels of CXCL-8 and decreased MCP-1 indicates altered immune responses. The combined levels of these two cytokines can be used to determine the risks of disease progression. The increase in plasma CXCL-8 and decreased MCP-1 levels were concluded based on the violin plots constructed. The combination of CXCL-8 and MCP-1 association in increasing the risk of LTBI by 14 folds was based on the binary regression analysis.

------------------------------------------------------------------------------------------------------

**Reviewer #2:**

**1. The manuscript by Selvavinayagam et al. presents the results of multiplex assessment of cytokines/chemokines of three cohorts of participants: household tuberculosis contacts with IGRA positive, IGRA negative result, and healthy controls. The assessment demonstrated significant differences in plasma concentration of two cytokines (CXCL8 and MCP-1) which makes them potential biomarkers of latent tuberculosis infection.**

**Reply:** Thank you for the recommendation.

**2. The results are interesting as many attempts to identify non-sputum-based biomarkers of tuberculosis infection and disease are being made. It is great to see two more potential biomarkers, however, the problem of this type of studies is that once the biomarkers are identified, they need to be tested on prospective cohorts of individuals to be validated. This is often lacking. I am wondering what the authors of the manuscript would suggest in terms of validation of identified biomarkers and their clinical application.**

**Reply:** We completely agree with the reviewer. The biomarkers identified herein will be tested and validated using prospective cohorts. Attempts are being undertaken to test and validate their likelihood for use in the field.

**3. As for the study design, my concern is how the study cohorts were formed. In some cohorts all participants were included, in others – only half of available participants. Why was it random selection and how can you be sure that there was no selection bias?**

**Reply:** Because the study consisted of three cohorts and that a limited number of samples could be run, we randomly selected the samples to be tested for the Bio-Plex cytokine assay. Referring to the table below, there is no significant different is all the demographics between the “selected” and “non-selected” participants indicating that there is no selection bias.

| **Demographic** | **Selected** | **Non-selected** | **P-value** |
| --- | --- | --- | --- |
| **Age,** year*;* *median (IQR)* | 36.5 (26.25 – 50.75) | 40 (32 – 50) | 0.224 |
| **Gender,** *n (%)* | 17 (30.4%) | 19 (34.5%) | 0.639 |
| **BMI,** *median (IQR)* | 24.33 (21.46 – 29.30) | 25.92 (22.95 – 28.89) | 0.301 |
| **BCG,** *n (%)* | 44 (78.6%) | 43 (78.2%) | 0.960 |
| **Residential area,** *n (%)* | 35 (62.5%) | 28 (50.9%) | 0.220 |

**4. Figure 2: not sure if some of these characteristics should be presented in Figure as they seem unrelated to LTBI. Usually the demographic data are presented as a table.**

**Reply:** The figure has been moved to supplementary (Supplementary Figure 1).

**5. I would also suggest to consider to re-formatting this manuscript as a short research letter rather than a full-text research article.**

**Reply:** We agree with the reviewer although the senior authors feel that this would dilute the vigour of the study.
