# Supplementary material for: Plasma CXCL8 and MCP-1 as surrogate plasma biomarkers of latent tuberculosis infection among household contacts–A cross-sectional study
Source: PLOS Glob Public Health. 2023 Nov 22;3(11):e0002327. doi: 10.1371/journal.pgph.0002327 (PMC10664947; doi:10.1371/journal.pgph.0002327)
Supplement: Attachment — Submitted filename: Biomarkers of LTBI among HHC _PLOS_Bijulal Octber 2023.docx [file pgph.0002327.s003.docx]

Thank you for this manuscript – which adds to the literature of studies aimed at detecting a biomarker signature to differentiate LTBI from none LTBI; where a strength is by including IGRA positive and negative active tuberculosis household contacts (and IGRA negative healthy controls).

1. In the methodology, it is not clear how the 172 participants were selected, and how the study population was selected from the 172 individuals. Also, how was active TB diagnosed among the index of the HHC – was the TB disease severity of comparable. How close were the HHC – as spouses tend to have higher contact, whereas teen age and older HHC may have less actual close contact in a home. How adequate was the sample size to significantly detect the differences?

2. It’s not clear what the rationale was for including the biochemical parameters. In fact, in the results – some trends were pointed out – would these differences be relevant in the interpretation of the biomarker results?

3. Looking at the figure 2 results, they were a lot of overlap in the results. What was the sensitivity, specificity, and predictive values of the individual significant markers?

4. In Section 3.3 – it is stated that “Our analysis showed that CXCL8 and MCP-1 could predict LTBI with the area under the curve (AUC) of 0.6885; p=0.0210 and 0.7392; p=0.0034, respectively.”.

However, in actual fact, figure 3A, the AUC shown was for either cytokine, not for both. The ROC for CXCL8 OR MCP-1 in figure 3 is stated as 0.9393. In the text it is changed to CXCL8 AND MCP-1 – which is a more likely interpretation. This needs to be clarified in the figure. In fact, the title could be changed to show that a 2 biomarker signature has high sensitivity in detecting IGRA positive HHC from IGRA negative HC. Also it would be of interest to test this 2 marker bio signature between HHC/IGRA pos compared to HHC/IGRA neg AND HC/IGRA negative – in addition to the age sub analysis – as this will be clinically of interest (especially where we find persistent IGRA negative HHCs.

5. This study is unique, as it noted a decrease in a biomarker MCP-1 a suggestive of LTBI, unlike most studies which concentrate on elevated markers. I suggest this should be indicated in the title.

6. Finally whereas the results and discussion sections were well written, the introduction and methodology sections rite up and grammar need to be edited, including the many short sentences.

10.

The authors need to discuss the possible mechanisms of the differences between the HHC/IGRA negative and the HC IgRA negative subjects
